# Editorial: Genetics and Genomics to Enhance Crop Production, Towards Food Security

**DOI:** 10.3389/fgene.2021.798308

**Published:** 2021-11-23

**Authors:** Ajay Kumar, Reyazul Rouf Mir, Deepmala Sehgal, Pinky Agarwal, Arron Carter

**Affiliations:** ^1^ Department of Plant Sciences, North Dakota State University, Fargo, ND, United States; ^2^ Sher-e-Kashmir University of Agricultural Sciences and Technology of Kashmir (SKUAST-K), Sopore, India; ^3^ International Maize and Wheat Improvement Center (Mexico), Texcoco, Mexico; ^4^ National Institute of Plant Genome Research (NIPGR), New Delhi, India; ^5^ Department of Crop and Soil Sciences, Washington State University, Pullman, WA, United States

**Keywords:** abiotic stress, disease resistance, candidate genes, association mapping, QTL mapping, genomic selection, gene editing, crop improvement

Twenty first century agriculture faces many challenges including new emerging abiotic and biotic stresses and decreasing arable land. These challenges pose serious threats to food security of an ever-increasing world population. One of the solutions to meet the food demands is to develop high-yielding crop varieties with greater genetic potential and resistance/tolerance to both biotic and abiotic stresses. Just like in mid-nineteenth century, when new genes and methods resulted in the first green revolution, there is a need to combine traditional plant breeding tools with new technologies to bring another green revolution for future food security.

In the last 3 decades, many new genomics tools and technologies (molecular markers, high density marker genotyping platforms, sequencing technologies, genome mapping methodologies, gene editing) have been developed, which have increased our knowledge and capabilities to breed improved varieties in a shorter period of time (Mir et al., 2013; Kumar et al., 2021; Sihag et al., 2021; Tyagi et al., 2021). The advances in genomics, statistical tools, bioinformatics, phenomics, data science and many other related disciplines are helping us achieve desirable genetic gains in crops, in a fast but sustainable manner (Mir et al., 2012; Mir et al., 2019; Salsman et al., 2018). To increase agricultural productivity to meet food demands, there is a need to combine traditional plant breeding tools and techniques with modern technologies like marker assisted/genomic selection and gene editing (e.g., CRISPR-Cas). Therefore, the focus of this topic was to bring together the research and knowledge of application of these modern tools for crop improvement.

This topic includes a total of 35 articles including 30 research articles and five review articles. The articles in this topic focus on a variety of new tools and technologies to develop knowledge and resources to breed high yielding, climate resilient crop varieties. This topic includes articles on genetic dissection of important traits related to yield, biotic and abiotic stress resistance, development of genomic tools to improve genomic predictions, cloning and deployment of important genes, and development of resources for enhancing crop production.

One of the major challenges in crop production is abiotic stresses. Among the abiotic-stresses, heat stress is considered one of the most important factors affecting yield in most crops, including cereals and pulses. This special topic includes several articles on focused on developing knowledge and genomic resources for breeding varieties tolerant to abiotic stresses (mainly drought tolerance) in important crops. In two different studies, Rabbi et al. used both genome-wide association mapping and quantitative trait locus (QTL) mapping combined with Infinium 90 K single-nucleotide polymorphism (SNP) assay to dissect the genetics of drought tolerance in hard red spring wheat (*Triticum aestivum* L.) in northern United States. In one of the studies, they evaluated 361 wheat genotypes, which were evaluated in nine different locations for yield and related traits under rain-fed conditions. Association mapping using mixed linear model identified a total of 69 consistent QTL with *p* ≤ 0.001. Six potential novel QTL were identified on chromosomes 3D, 4A, 5B, 7A, and 7B. The resources developed in this study could be used in marker-assisted selection for drought-tolerance breeding in spring wheat. In another study, Rabbi et al. used a wheat RIL population derived from a cross between a drought-tolerant cultivar and a high-yielding but drought-susceptible cultivar. They identified a total of 11 consistent QTL for drought tolerance-related traits; six QTL being exclusively identified in drought-prone environments, and five constitutive QTL (identified under both drought and optimal conditions). Major QTL expressed exclusively under drought environments were identified on chromosomes 7B and 2B, while a novel QTL for drought tolerance was identified on chromosome 2D. The authors also conducted *in silico* expression analysis of candidate genes located in the QTL regions. They identified the enrichment of ribosomal and chloroplast photosynthesis-associated proteins showing the most expression variability, thus possibly contributing to stress response. The significant QTL identified in this study will play an important role in the development of drought-tolerant wheat cultivars using genomics-assisted breeding approaches.

In rice (*Oryza sativa* L.), Chen et al. identified a new major QTL for heat tolerance during the flowering stage using an F_2:3_ population. The QTL *qHTT8* was localized to chromosome 8 of rice and contained 65 predicted genes, 10 of which were found to be associated with abiotic stress tolerance. Using qRT-PCR, the differential expression of these 10 putative genes under high temperature conditions was conducted, and it was found that *LOC_Os08g07010* and *LOC_Os08g07440* were highly induced in the heat tolerant cultivar. These results will be used to develop heat tolerant rice cultivars, as well as future efforts to clone *qHTT8* to understand its functionality.

Chickpea (*Cicer arietinum* L.) is one of the most important legumes crops in the world and this crop is mainly grown in rainfed conditions under residual moisture and restricted irrigation. The comprehensive study by Kumar et al. used comparative study of a candidate gene, molecular marker, and physiological traits for screening drought tolerance in chickpea. The abiotic stress-responsive gene “Dehydrin (DHN)” was also identified based on a sequence similarity approach. Most promising drought tolerant genotypes (Pusa1103, ICC4958 and Pusa362) were identified in this study and will prove useful in future chickpea breeding programs aimed at enhancing drought tolerance. In another research article by Meena et al., a set of six members of the *TaSTI* family were identified and among them “TaSTI-2” members were found to express higher as compared to “TaSTI-6” members under heat stress conditions, with *TaSTI-2A* being the most heat responsive member. They showed for the first time that TaSTI-2A interacts with TaHSP90 not only in the nucleus but also in the endoplasmic reticulum and Golgi bodies.

Several articles also focused on genetic dissection of disease resistance and development of genomic resources for molecular breeding. One of the articles focused on identification of novel QTL for spot blotch caused by *Bipolaris sorokiniana,* which is one of the major diseases of wheat under tropical and subtropical conditions (Tomar et al.). Using 139 advanced spring wheat lines and 14,063 polymorphic genotyping-by-sequencing (GBS) markers, Tomar et al. identified eight QTLs associated with spot blotch disease resistance in wheat. The authors also conducted functional annotation of the significant markers, which identified NBS-LRR, MADS-box transcription factor, and 34 other plant-related protein families distributed across the genome. This study identified four new QTLs on chromosomes 1A (497.2 Mb), 1D (89.84 Mb), 2B (421.92 Mb), and 6D (6.84 Mb) associated with several disease resistance protein families. The results of this study could be vital for spot blotch resistance breeding in wheat.

With an objective to transfer white mold resistance (caused by *Sclerotinia sclerotiorum*) into an adapted pea (*Pisium sativum* L.) background, Mahini et al. developed and evaluated two RIL populations (Lifter × PI240515 and PI169603 × Medora) for resistance to white mold by measuring lesion expansion inhibition (LEI) and nodal transmission inhibition (NTI). This study identified a total of 22 short genotypes with partial resistance. Using GBS-derived linkage maps and inclusive composite interval mapping, they identified a total of thirteen QTL associated with white mold resistance traits in both populations. Three of them were co-located with height genes and the other ten QTL were associated with LEI (7) and NTI (3). The resistant lines and genetic resources developed in this study will help accelerate the development of white mold resistance cultivars using molecular breeding approaches.

Common bean (*Phaseolus vulgaris* L.) is considered one of the principal grain legume crops of western-Himalayas of Jammu and Kashmir (Choudhary et al., 2018a; Choudhary et al., 2018b). Huge diversity exists in common bean germplasm from this region. A core set of 96 genotypes selected from a set of 428 genotypes were selected for study of allelic diversity, structural analysis, and genome wide association study (GWAS) for yield and related traits using unexplored common bean germplasm from the western Himalayas (Mir et al.). The study of allelic diversity led to the identification of 691 alleles ranging from 2 to 21 with an average of 7.59 alleles/locus. GWAS for pods/plant, seeds/pod, seed weight, and yield/plant led to the identification of 39 marker-trait associations (MTAs) including 15 major, 15 stable, and 13 both major and stable MTAs. These MTAs will prove useful for common bean breeding programs world-wide.

Breeding disease resistant wheat varieties is one of the most important subject areas of wheat research and wheat blast (*Magnaporthe oryzae* pathotype *Triticum*) is considered one of the most destructive diseases in South American and South Asian countries. GWAS was conducted using a diverse panel of 184 wheat genotypes from South Asia and CIMMYT and genotyping data of 11,401 SNP markers of the Illumina Infinium 15 K BeadChip (He et al.). This study led to the identification of MTAs on chromosomes 1BS, 2AS, 6BS, and 7BL. The most promising resistant accessions/genotypes identified in this study will prove useful as a preemptive strategy to prevent wheat blast outbreaks in South American and South Asian countries.

Breeding for wheat stripe (yellow) rust (*Puccinia striiformis* f. sp. *tritici*) is one of the most important subject areas of wheat research. Therefore, efforts have been made to characterize wheat germplasm for novel sources of resistance and their incorporation into elite cultivars. GWAS was conducted using a set of 391 genotypes and 35 K Axiom^®^ array with the aim to identify QTL for stripe rust resistance at the seedling stage (Pradhan et al.). A total of 40 QTLs were identified, of which 20 QTLs were found to be closely linked with previously reported stripe rust resistance genes/QTLs on chromosomes 1B, 2B, 5B and 6B, whereas the 20 novel QTLs were mapped on chromosomes 2D, 3A, 3D, 5A and 7D. These 20 novel QTL identified in the present study might play a key role in marker-assisted breeding for developing stripe rust resistant wheat cultivars (Pradhan et al.).

Zitnick-Anderson et al. conducted a GWAS on the Andean and Middle American diversity panels of common bean to determine the genetic basis of resistance to Fusarium root rot (*Fusarium solani*) using a greenhouse screening assay developed by them. Important associations were identified in both panels harboring important candidate genes with proven roles in plant defense including Glucan synthase-like protein, NAC domain protein, senescence-associated genes, MAC/Perforin domain-containing gene, and ethylene response factor 1. Most importantly, it was discovered that some common genetic factors might be present to Pv02, Pv09 and Pv11 in the Andean gene pool and to Pv01 and Pv08 in the Middle American gene pool that might be involved in the resistance of both Rhizoctonia and Fusarium root rot in the common bean.

Ranganatha et al. conducted QTL mapping to determine genomic regions controlling resistance to northern corn leaf blight (*Exserohilum turcicum*) in maize (*Zea mays* L.) and identified a large effect QTL on chromosome 8 explaining the highest percentage variation of 16.34%. QTL for two more foliar diseases [sorghum downy mildew (*Peronosclerospora sorghi*) and southern corn rust (*Puccinia polysora*)] were also identified on chromosomes 2 and 10 in order to generate pyramided lines expressing QTL for resistance to all three foliar diseases. Markers linked to these major QTL were deployed in marker-assisted selection and 125 pyramided lines were developed, of which 39 lines exhibited an acceptable level of resistance to the three major diseases.

The net blotch of barley (*Hordium vulgare* L.) caused by the necrotrophic fungal pathogen *Pyrenophora teres* f. *teres* (*Ptt*) has become a serious threat to barley production globally. Tamang et al. employed a mutagenesis approach to identify resistance genes present in barley line CI_5791_. Seeds of CI_5791_ were irradiated with γ-rays and an M_2_ population was generated from which two mutants, CI_5791-γ3_ and CI_5791-γ8_, were sequenced by exome capture. Comparison of their sequences with wild-type CI_5791_ identified independent mutations in the *HvWRKY6* transcription factor located on chromosome 3H, which suggested *HvWRKY6* as a strong candidate gene. Gene silencing experiments conducted in barley line CI_5791_ confirmed the hypothesis that *HvWRKY6* is responsible for imparting resistance to net blotch.

*Pyrenophora tritici-repentis*, causing tan spot disease in wheat, is a serious threat to wheat production in Kazakhstan. Kokhmetova et al. conducted a GWAS in a collection of spring and winter wheat lines originating from Kazakhstan, Russia, and CIMMYT breeding programs to determine the genetic basis of resistance to tan spot. The most significant finding of this study was the identification of a 3 Mb genomic region on chromosome 6A, where SNPs associated with resistance to the two most prevalent races, Race 1 and Race 5, were identified. This study has opened vistas to initiate molecular marker-assisted breeding activities in Kazakhstan and twenty-five lines carrying novel combinations are being used in the country’s breeding programs to improve resistance against both races.

Because of importance of plant roots for water and nutrient acquisition, environmental adaptation, and yield formation, Xu et al. used a total of 196 wheat accessions from the Huang-Huai Wheat Region of China to investigate six root traits. This study showed that the root traits varied most under outdoor pot culture, followed by those under both outdoor hydroponic culture and indoor hydroponic culture, and root elongation under hydroponic culture was faster than that under pot culture. A GWAS using the Wheat 660 K SNP Array identified 12 stable chromosomal regions associated with root traits located on chromosomes 1D, 2A, 4A, 4B, 5B, 6D, and unmapped markers. Linkage disequilibrium blocks identified 27 genes related to root development including *TraesCS4A02G484200, TraesCS4A02G484800*, *TraesCS4A02G493800*, and *TraesCS4A02G493900*, which were involved in cell elongation and differentiation.

Three important articles in this topic focused on development of genomic tools for yield and yield components in wheat. Sehgal et al. reported a comprehensive GWAS and epistatic interactions analysis to investigate the genetic architecture of grain yield (GY) using a large set of 6,461 advanced breeding lines from the CIMMYT breeding program. The authors used a linkage disequilibrium approach to generate 519 genome-wide haplotype blocks and used them in combination with GY data generated under irrigated and stress environments to conduct a haplotypes-based GWAS and analysis of epistatic interactions. In addition to seven hotspots regions reported on chromosomes 1A, 1B, 2B, 4A, 5, 6, and 7B controlling GY, the authors unveiled that a large proportion of the variation was explained by epistatic interactions. To utilize these results for breeding application, the authors constructed heat maps and identified sets of lines having multiple QTL to be included in crossing schemes.

Cao et al. conducted QTL mapping for thousand grain weight (TGW), grain width (GW), and grain length (GL) in wheat using a RIL population derived from the cross between Jing 411 and Hongmangchun 21. The authors not only identified five new stable QTLs for TGW and GL on chromosomes 1B, 2A, 4B, and 7A, but also validated their effects in an independent Chinese mini core collection (CMCC). In addition, *in silico* analysis of the stable QTL in the IWGSC annotation database predicted important candidate genes including *TraesCS4B02G376400* encoding a plasma membrane H^+^-ATPase. Investigation of allelic variation in this gene in the CMCC showed significant association of a SNP with TGW, GW and GL.

In order to better understand hybrid wheat seed production, Adhikari et al. performed GWAS and genomic prediction of anther extrusion in the CIMMYT hybrid wheat breeding program. A set of 603 male lines were phenotyped across three field experiments and genotyped with the 20 K Infinitum iselect SNP Array. GWAS produced five MTA, each with only a small effect. Genomic prediction involved the main effects of lines, environments, genomic and pedigree relationship, as well as their interactions in different statistical models, which were evaluated in three cross-validation scenarios. Genomic prediction appeared to be a very reliable tool for predicting anther extrusion in hybrid wheat breeding, and could be used in selection across breeding cycles, aiding in rapid breeding for this trait.

This research topic Research Topic also includes a few articles in vegetable crops, like Chinese cabbage (*Brassica rapa* L. spp. *Pekinensis*), *Capsicum*, and European turnip (*Brassica rapa* L. ssp. *rapifera*). An important trait in Chinese cabbage is leaf adaxial-abaxial polarity as it relates to the formation of leafy heads. Therefore, Gao et al. identified genes responsible for this trait, as well as their genetic variation, in order to further understand the mechanism of leafy head formation. A total of 41 candidate genes were identified through comparisons with *Arabidopsis thaliana*, which lead to further understanding of gene structure. Based on these 41 candidate genes, 341 simple sequence repeats were detected, and 323 loci were used to design 969 specific primers. A subset of these primers were selected randomly and evaluated using 12 Chinese cabbage accessions with different heading types. A total of 23 new primer pairs were combined with 127 other markers to construct a linkage map from an F_2_ population of two cabbage parents with different heading types. These new markers will continue to assist in the understanding of traits associated with the formation of leafy heads in Chinese cabbage as well as assist in the breeding and selection of this trait.

The article by Luo et al. reported a systematic identification and expression-characteristic analysis of *CaNHX* genes at the whole genome level in cultivated and wild pepper (*Capsicum annuum* L.). The study identified a total of 42 CaNHX genes, with nine having complete functional domains of the Na^+^/H^+^ exchanger gene. The authors also conducted transcriptome analysis which showed that *CaNHX* genes were upregulated under various abiotic stresses. The Na^+^/H^+^ exchangers (NHXs) play an important role in plant growth and abiotic stress tolerance. Therefore, the findings of this study could play a vital role in breeding stress tolerant pepper.

Park et al. used single-molecule long-read sequencing technology to generate a draft genome assembly of a European turnip, ECD4. This line has strong clubroot resistance, a disease caused by *Plasmodiophora brassicae*. The authors believe that the draft assembly and transcriptome data developed in this study will help the development of genetic and genomic resources for *Brassica* improvement, including the identification of clubroot and other disease resistance genes (*R* genes), the study of diversity and evolution, and markers for genomics assisted breeding in *Brassica.*

Few articles in this topic also focused on transcriptome analyses to identify important candidate genes. In *japonica* rice, *GW2* is a previously identified QTL known as being a negative regulator of grain width. The related SNP is absent in *indica* rice. Verma et al. knocked down *GW2* in *indica* rice and reported increases in grain width, length and weight. This was because of a decrease in cell size but an increase in cell number. The phenotype was stable across three generations of seeds. The starch granules in the endosperm were bigger in size, tightly packed, and showed an increase in polyhedral granules in comparison to the wild type. Transcriptome analyses of seeds from a homozygous line and its comparison with wild type seeds showed 1,426 differentially expressed genes (DEGs), of which 55 have been previously characterized to be involved in rice seed development. Comparison of the DEGs from this analysis with DEGs from seed development transcriptome resulted in 23 seed specific DEGs which were commonly upregulated in both tissues. This implies that *GW2* works upstream of these seed-related genes and regulates their expression.

Elongation of rice peduncles prior to heading determines panicle exsertion, which in turn is directly related to panicle fertility, and hence, yield. Kandpal et al. performed transcriptome analyses on two Indian cultivars contrasting for peduncle elongation. Tissue used for the analyses were elongating peduncles (EP) and non-elongating peduncles (NP) from Swarna (with lesser peduncle elongation) and Pokkali (with higher peduncle elongation). EP of both cultivars had 1,500 common up regulated genes while NP had 1723 genes. Genes differentially expressed in EP coded for cellulase synthases, sucrose synthases, invertases, sucrose transporters, auxin and brassinosteroid biosynthesis and signaling, and 836 transcription factors. Also, 50 genes that were up-regulated in EP were also involved in pollen formation. The authors conclude that the data generated in their study can be used further for improving peduncle elongation in rice.

Wang et al. reported genome-wide identification and capsaicinoid biosynthesis-related expression analysis of the R2R3-MYB gene family in *Capsicum annuum*. Capsaicinoids are naturally specialized metabolites and main factor causing *Capsicum* pungency. The MYB transcription factors (R2R3-MYB subfamily) are key regulators and candidate factors in capsaicinoid biosynthesis. The authors identified a set of 108 R2R3-MYB genes and conducted interspecies collinearity analysis which revealed that the R2R3-MYB family contains 16 duplicated gene pairs. The highest gene density was observed on chromosome 00 and 03. The *CaR2R3-MYB* genes were differentially expressed, and capsaicinoid-biosynthetic genes (CBGs) in fruit development stages were obtained via RNA-seq and qRT-PCR. The study further revealed that a set of six candidate *CaR2R3-MYB* genes are involved in regulating the synthesis of capsaicin and dihydrocapsaicin.

Hou et al. also performed transcriptome analyses to understand an industrially important species, *Gnetum luofuense*. The tissues used were four developmental stages (GLN01-04) of the stem apex from female and male plants. Amongst the expressed genes, the terms ribosome, spliceosome and carbon metabolism showed the highest hits in the KEGG database. In conjunction, 24,151 alternative splicing events were detected, in which intron retention was the highest. Also, 38,108 complete open-reading frames and 728 lncRNAs were identified. Transcription factors and their putative pathways were also identified from the transcriptome data. Using K-means clustering, differentially expressed transcripts amongst the four tissues were delineated. Most differentially expressed transcripts were present between GLN01 and GLN04, which belonged to phenylpropanoid biosynthesis, starch and sucrose metabolism, and cyanoamino acid metabolism. The data generated in this study is a compendium of transcriptome changes in stem apex development in *Gnetum*.

Williamson-Benavides et al. have identified differentially expressed genes in *Pisum sativum* in response to *Fusarium solani* infection, which causes root rot. The tissues used were four tolerant and four susceptible pea genotypes, in a time-course experiment (0, 6 and 12 h). In response to *Fsp* infection, 42,905 differentially expressed contigs (DECs) were identified. The susceptible genotypes had more DECs upon infection. As with other transcriptome analyses, all DECs were subject to functional annotation and GO enrichment. The study concluded that DECs could be categorized into seven broad categories including transcriptional regulation, pathogenesis-related, anthocyanin and lignin biosynthesis, phytohormone, cell wall and membrane metabolism, and toxin metabolism. The analyses highlight genes which can be targeted to tackle pea root rot. Further, in another study, Williamson-Benavides et al. delineated QTLs for pea root rot resistance from the DEGs identified in the above study. Two RIL populations (190 individuals each) were used to identify QTLs using composite interval mapping. This resulted in 47 SNP markers. Out of these, five were associated with root disease severity and height. This data was overlaid with the transcriptome data mentioned above. Eventually, seven, 17, 22, 14, and 11 DEGs were identified in different genotypes to be associated with disease response. The study identified a major and four minor QTLs, using a combination of transcriptome analyses and QTL mapping. The underlying genes can further be used for imparting root rot resistance in pea.

Following discovery of genes and QTLs, the next important step in crop improvement is their validation and transfer into different genetic backgrounds and development of improved varieties. In this direction, the study by Kaur et al. pyramided genes of grain weight, stripe rust, and leaf rust (*Puccinia triticina*) into elite Indian bread wheat cultivars (PBW343 and PBW550) using both marker-assisted selection and phenotypic selection. The results showed an increase in grain weight of 8.82–10.27 g in PBW343 and 5.27–7.6 g in PBW550 through conventional backcross breeding and phenotypic selections. The improved genotypes in the PBW550 background possess increased grain weight ranging from 45.0 to 46.2 g and three stacked genes for stripe and leaf rust resistance, which is a valuable resource for breeding elite cultivars.

Traditional marker assisted selection incorporates few markers in the selection scheme and is mostly limited to selection of few genes/QTL with major effects. However, the majority of important traits like yield and abiotic stresses have complex genetic architecture and are controlled by many genes. To expedite the genetic gain for such traits, the concept of whole genome prediction based selection (or genomic selection) is gaining momentum. The study by Tessema et al. attempted to quantify the increase in genetic gain by implementing genomic selection in traditional wheat-breeding programs. They studied the effect of genetic correlation between different traits on genetic gain. The study reported three times higher genetic gain using genomic estimated breeding values compared to phenotypic selection, suggesting the importance of genomic selection for improving genetic gain in wheat-breeding programs. This study showed increased genetic gain when genomic information is incorporated in a conventional breeding program.

The article by Cuevas et al. focused on improving genomic prediction models. Genomic prediction and selection are becoming important tools in both animal and plant breeding. However, these genomic tools encounter several computational challenges when the number of observations (*n*) is large. Inclusion of genomic × environment interaction and multi-trait kernels in the prediction model further complicates the computational capabilities. The study by Cuevas et al. proposed selecting a small number of lines *m* (*m* < *n*) for constructing an approximate kernel of lower rank than the original. Doing so exponentially decreased the required computing time. When the authors applied the proposed method to two different wheat data sets of different sizes (*n*) using the standard linear kernel Genomic Best Linear Unbiased Predictor (GBLUP) and also using eigen value decomposition, results showed a competitive prediction performance of the approximated methods with a significant reduction in computing time.

This Research Topic also included few review articles focused on application of modern phenomic and genomic based tools for addressing challenges to crop production. For examples, Jha et al. reviewed the progress made in developing genetic and genomic resources for Fusarium wilt (Fusarium oxysporum), whichis the key constraint to grain legume production worldwide. This review article provides a brief recap of classical genetic efforts, as well as the latest technological breakthroughs that have enhanced our understanding of the genetic basis of both plant resistance and pathogenicity. This article presented several examples where modern functional genomic tools like RNA-seq, proteomics, and metabolomics are playing a greater role in illuminating the various aspects of plant-pathogen interaction. At the end, they discussed future prospect for breeding for *Fusarium* wild resistance in grain legumes including the application of modern high throughput phenomics and genomics-based breeding techniques to develop *Fusarium* wild resistant grain legumes.

Grain number is one of the major yield components in cereals and thus important to enhance grain yield. In rice, grain number has been found associated with shoot branching (tillering), panicle branching, panicle length, and seed set percentage. It has also been shown that all these traits are controlled by phytohormones by regulating molecular factors and their cross-interactions. This review by Deveshwar et al. focuses on the molecular machinery, involving several genes and QTL, operational in the plant that governs hormonal control and, in turn, gets governed by the hormones to regulate grain number and yield in rice.

An important review on the development of genetic and genomic resources for molecular breeding and technological advances, especially in the area of genome editing, in cassava (*Manihot esculenta* Crantz) was compiled by Mbanjo et al. The authors also reviewed various phenotyping protocols that have been used to phenotype cassava germplasm collections for important agronomic traits including nutritional traits (carotenoids, cyanogenic potential), dry matter content (substitute for yield), quality traits, root architecture, and resistance to multiple diseases (cassava mosaic virus, brown streak disease, bacterial blight, root rot). An update was also provided on how outputs of gene discovery and genomic selection studies have been practically utilized in crop improvement.

Saad et al. reviewed the literature for genomics approaches in *Brassica* improvement in a changing global environment. The article reviewed the literature on genetic diversity, genomic resources in *Brassica*, nuclear genome, organelle genomes, marker discovery in *Brassica*, epi-genomics, reverse genetics, genome manipulation, genome editing, transcriptomics, transformation, and genomic selection in crop improvement.

The article by Verma et al. reviews the importance of studying plant DNA repair and recombination (DRR) for crop improvement. The knowledge about plant DRR could help design new strategies for further crop improvement to meet the demand of billions of humans in a sustainable environment. In plants, there are different DRR mechanisms including direct repair, nucleotide excision repair, base excision repair, mismatch repair, non-homologous end joining, and homologous recombination, which interact to continuously repairs DNA damages. In this review, the authors provide an overview of different DRR pathways, their structural and biochemical aspects, and their potential for crop improvement.

In summary, this topic comprises of a diverse collection of both original research and review articles. These articles generated a large amount of genetic and genomic resources in different crops including major cereals (wheat, rice, maize, barley), legumes (chickpea, pea, common bean), and vegetable crops (Chinese cabbage, *Capsicum*, European turnip). The articles in this topic reported the application of many modern genomic tools for crop improvement, like QTL mapping, GWAS, high density marker platforms in wheat (Infinium 90 k and 35 K Axiom^®^ array), genotype by sequencing, marker assisted and genomic selection, single-molecule long-read sequencing technology, transcriptome analyses, candidate gene analyses, etc. We believe that the resources and knowledge generated through articles published in this topic will help expediting crop improvement, thus playing an important role in increasing crop production for future food security.

## References

[B1] Choudhary N. Bawa V. Paliwal R. Singh B. Bhat M. A. Mir J. I. (2018b). Gene/QTL Discovery for Anthracnose in Common Bean (*Phaseolus vulgaris* L.) from North-western Himalayas. Plos One 13, e0191700. 10.1371/journal.pone.0191700 29389971 PMC5794095

[B2] Choudhary N. Hamid A. Singh B. Khandy I. Sofi P. A. Bhat M. A. (2018a). Insight into the Origin of Common Bean (*Phaseolus vulgaris* L.) Grown in the State of Jammu and Kashmir of north-western Himalayas. Genet. Resour. Crop Evol. 65, 963–977. 10.1007/s10722-017-0588-z

[B3] Kumar S. Kumar M. Mir R. R. Kumar R. Kumar S. (2021). “Advances in Molecular Markers and Their Use in Genetic Improvement of Wheat,” in Physiological, Molecular, and Genetic Perspectives of Wheat Improvement. Editors Wani S. H. Mohan A. Singh G. P. (Cham, Switzerland: Springer Nature), 139–174. 10.1007/978-3-030-59577-7_8

[B4] Mir R. R. Hiremath P. J. Riera-Lizarazu O. Varshney R. K. (2013). “Evolving Molecular Marker Technologies in Plants: from RFLPs to GBS,” in Diagnostics in Plant Breeding. Editors Lübberstedt T. Varshney R. K. (New York: Springer), 229–247. 10.1007/978-94-007-5687-8_11

[B5] Mir R. R. Zaman-Allah M. Sreenivasulu N. Trethowan R. Varshney R. K. (2012). Integrated Genomics, Physiology and Breeding Approaches for Improving Drought Tolerance in Crops. Theor. Appl. Genet. 125, 625–645. 10.1007/s00122-012-1904-9 22696006 PMC3405239

[B9] Mir R. R. Reynolds M. Pinto F. Khan M. A. Bhat M. A. (2019). High-Throughput Phenotyping for Crop Improvement in the Genomics Era. Plant Sci. 282, 60–72. 31003612 10.1016/j.plantsci.2019.01.007

[B6] Salsman E. Kumar A. AbuHammad W. Oladzad-Abbasabadi A. Dobrydina M. Chao S. (2018). Development and Validation of Molecular Markers for Grain Cadmium in Durum Wheat. Mol. Breed. 38, 28. 10.1007/s11032-018-0788-z PMC5844312 29352079

[B7] Sihag P. Sagwal V. Kumar A. Balyan P. Mir R. R. Dhankher O. P. (2021). Discovery of miRNAs and Development of Heat-Responsive miRNA-SSR Markers for Characterization of Wheat Germplasm for Terminal Heat Tolerance Breeding. Front. Genet. 12, 699420. 10.3389/fgene.2021.699420 34394189 PMC8356722

[B8] Tyagi S. Kumar A. Gautam T. Pandey R. Rustgi S. Mir R. R. (2021). Development and Use of miRNA-Derived SSR Markers for the Study of Genetic Diversity, Population Structure, and Characterization of Genotypes for Breeding Heat Tolerant Wheat Varieties. PLoS One 16, e0231063. 10.1371/journal.pone.0231063 33539339 PMC7861453

